# Time and safety in defibrillation with paddles versus pads; a comparative study of two defibrillation regimes

**DOI:** 10.1186/1757-7241-20-S2-P50

**Published:** 2012-04-16

**Authors:** Benedikte Møhl Halle, Henriette Ullerup-Aagaard, Lars Folkestad, Mikkel Brabrand

**Affiliations:** 1 Department of Medicine, Esbjerg Hospital, Denmark; 2 Department of Cardiology, Odense University Hospital, Denmark

## Background

New resuscitation guidelines were published in October 2010. These recommend use of pads instead of paddles and recommend that CPR is continued during charging of the defibrillator.

The aim of our study was to investigate the difference in time spent until delivery of the first and second shock using pads versus paddles, and to assess the rescuers subjective feeling of safety when continuing CPR while charging the defibrillator.

## Methods

We asked the participants to defibrillate two porcine thoraxes following 2010 guidelines. One was dry, the other wet to simulate sweating.

All participants defibrillated both thoraxes using paddles and pads. We used Defib-Pads (3M Health Care) for paddles, using a HpCodemaster (HewlettPackard). For pads we used Quick-Combo electrodes (Medtronic) and a LifePak 20 (Medtronic).

We recorded time to delivery of the first shock. After two minutes of CPR, time to delivery of the 2nd shock was recorded. Burn marks and whether the pads or gel pads had moved was also noted.

Participant preferences and prior experiences were collected.

Statistics were calculated using Stata 11.2 (StataCorp) using Pearson’s Chi-squared and Wilcoxon’s rank sum test.

## Results

9 junior doctors were included, 6 had participated in cardiac arrest treatment.

First shock on a dry surface was delivered in median 30.3 seconds using pads, and in median 21.5 seconds with paddles, p=0.06.

On a wet surface it was delivered in median 31.6s with pads, and in median 18.5s using paddles, p<0.01.

Second shock on a dry surface was delivered in median 159s using pads, and in median 150s with paddles, p=0.1.

On a wet surface the 2nd shock was delivered in median 162.2s with pads, and in median 149s using paddles, p=0.02.

The participants preferred pads regarding safety (p<0.01). Only one shock (paddles on a wet thorax) gave a burn mark. No difference in replacement of pads or paddles was recorded.

## Conclusion

Time to first shock was significantly shorter using paddles. Time to second shock was non-significantly shorter using pads. The participants felt safer using pads.

